# Deconstructing Gender Differences in Experienced Well-Being Among Older Adults in the Developing World: The Roles of Time Use and Activity-Specific Affective Experiences

**DOI:** 10.1007/s11205-020-02435-3

**Published:** 2020-07-24

**Authors:** Gabriela Flores, Clémence Kieny, Jürgen Maurer

**Affiliations:** Faculty of Business and Economics, University of Lausanne, Internef, Chamberonne, 1015 Lausanne, Switzerland

**Keywords:** Experienced well-being, Subjective well-being, Day reconstruction method (DRM), Gender, Older adults, Low- and middle-income countries

## Abstract

Due to declining fertility rates and increasing longevity, the world is growing older. Improving the quality of life of older adults, and not merely preventing deaths, is thus becoming an important objective of public policies. It is, therefore, urgent to understand the key dimensions of older adults’ subjective well-being as well as their main drivers. Women represent a large proportion of the older population, and existing evidence suggests that they may be particularly vulnerable, especially in the developing world. Analyzing potential gender differences in experienced well-being in older adults is hence crucial. We exploit information on time use and activity-specific emotional experiences from the abbreviated version of the day reconstruction method contained in the WHO Study on Global Ageing and Adult Health (SAGE), focusing on five developing countries. We first quantify gender differences in experienced well-being among older adults, which we then deconstruct into corresponding differences in time use and activity-specific net affects. Adjusting for age only, our results indicate a gender gap in experienced well-being in favor of men. Yet, adjusting for additional individual characteristics and life circumstances beyond age weakens this association. Illustrative counterfactual analyses further suggest that gender differences in activity-specific net affects appear more important than differences in time use for explaining the disadvantage of older women. Our results suggest that women’s lower affect in most activities is linked to the conditions under which these activities are performed, and in particular to the higher level of disability of older women compared to men of the same age.

## Introduction

Subjective well-being is increasingly recognized as an indispensable complement to traditional indicators of economic performance and human development, such as Gross Domestic Product (GDP) or Human Development Indices (HDI), to comprehensively assess and track the welfare of societies as a whole as well as the well-being of different population groups (Stiglitz et al. 2009; Dolan et al. 2008). Besides striving to increase performance in terms of health, economic outcomes, and education, governments should therefore also take into consideration the impact of institutions and policies on individuals’ subjective well-being. Arguably, policy-makers should at least in part be guided by the priorities of citizens themselves, and optimizing the subjective well-being of the population should thus constitute a meaningful policy objective in itself. Moreover, several studies suggest that subjective well-being may also influence more objectively measurable life circumstances, such as productivity and social behavior, as well as individuals’ health and longevity (e.g., De Neve et al. 2013; Diener et al. 2017), which further highlights the importance of subjective well-being as a key goal of health, social, and economic policies. Support for using subjective well-being assessments as policy indicators is based on growing evidence that self-reports of subjective well-being are a valid way to measure individual welfare and happiness. For example, several neuroimaging studies have shown that subjective well-being reports are closely related to multiple cognitive-emotional brain regions (e.g., Luo et al. 2014; Sato et al. 2015; Ren et al. 2019).

The age structure of the world population is changing due to declining fertility rates and increasing longevity. Worldwide, the share of the population over the age of 65 years has increased from 6 to 9 percent between 1990 and 2019 and is projected to increase to 16 percent by 2050, reaching a total of 2 billion individuals falling into this age group (United Nations 2019a). Understanding the drivers of subjective well-being in older adults and thus the potential impacts that global aging will have on the overall well-being of the society is thus essential (National Research Council 2013). In line with this new demographic reality, global institutions are increasingly acknowledging well-being in old age as an important issue: Indeed, the 2030 Agenda for Sustainable Development states the promotion of well-being at all ages as one of its goals (United Nations 2019b) and the World Health Organization (WHO) defines Healthy Ageing as “the process of developing and maintaining the functional ability that enables well-being in older age” (World Health Organization 2015). Yet, much of the academic discussion regarding the subjective well-being of older adults focuses on what is known from experiences in high-income countries, while little is known about the situation in low- and middle- income countries, where 80 percent of the worlds’ elderly will be living by 2050 (Shetty 2012).

Further examination of the current demographic situation reveals that the older population is predominantly female and is likely to remain so in the foreseeable future. On average, during the period 2010–2015, women outlived men by about 4.5 years. In 2017, women thus represented 54 percent of those aged 60 years and above and 61 percent of those aged over 80 (United Nations 2019c). However, although women may generally expect to live longer than men, there is evidence that they experience higher morbidity than men of the same age (e.g., Verbrugge 1985; Denton et al. 2004; Case et Paxson 2005). In addition, women’s lower participation in the paid workforce throughout their life^1^ bears negative consequences in older age, such as lower access to pensions and other economic resources resulting in greater poverty, lower access to healthcare and social care services, and higher risk of abuse (World Health Organization 2015). Finally, compared to men, women of all ages tend to spend more time on non-leisure activities such as unpaid housework (Miranda 2011). As highlighted in the 2012 World Development Report on Gender Equality and Development (World Bank 2012), in most countries, women allocate between one and three more hours per day to housework as compared to men, spend two to 10 times more hours on care-related activities, and up to four hours less on market activities. Several studies further analyze how discrepancies in the gender division of labor evolve over the life course. For example, the World Development Report (World Bank 2012) notes that the above patterns are often accentuated after marriage and childbearing but diminish with older age. While there are clear gender differences in time use, there is little evidence to date regarding the impact of these differences in time use on subjective well-being, especially on how men and women feel as they live their daily lives (experienced well-being).

Subjective well-being is a multifaceted concept, which is commonly divided into two constituent components: evaluative and emotional well-being (National Research Council 2013; OECD 2013^)^.^2^ Measures of evaluative well-being on the one hand are more commonly available for analysis and are based on respondents’ cognitive evaluations of their own life, often using questions such as “how would you rate your life overall these days”. Measures of emotional well-being, on the other hand, aim to capture respondents’ affective experiences as they live their lives such as feeling calm, relaxed, worried, stressed or angry. Boarini et al. (2012)—among others—argue that measures of evaluative and emotional well-being are both conceptually and empirically distinct: while life satisfaction seems to be more closely related to cognitive judgements of how individuals evaluate their own life and how they compare it to that of others, affective experiences seem to be strongly influenced by time use. In addition, Kahneman and Krueger (2006) claim that measures of emotional well-being may be less subject to individual reporting biases compared to measures of evaluative well-being. Similarly, Kahneman and Riis (2005) argue that measures of emotional well-being may be less influenced by cultural disposition, self-conceptualization, memory and introspection. Moreover, they emphasize that emotional well-being may be a more important determinant of future health due—for example—to the cumulative effects of stress. Consistent with this idea that the quality of peoples’ daily experiences is linked to health outcomes, several authors show that emotional well-being is a strong predictor of mortality (e.g., Carstensen et al. 2011; Steptoe and Wardle 2012). Specifically, experienced well-being is a duration-weighted measure of emotional experiences as people live their everyday lives. It thus records and aggregates emotional experiences through time to obtain a measure of individual emotional well-being. This approach to measuring well-being also has a long-standing tradition in economics: In 1881, Edgeworth proposed to record utility as the quality of experiences at every instant in order to create what he called a “*hedinometer*” (Edgeworth 1881). As formulated by McFadden (2005), Edgeworth “*envisioned the level of happiness associated with an experience as the integral of the intensity of pleasure over the duration of the event*” (p. 3).

Our study investigates gender differences in experienced well-being as a conceptualization of emotional well-being. Most of the literature on gender differences in subjective well-being focuses on evaluative well-being while studies examining gender differences in emotional well-being are scarcer (see for example Batz and Tay (2018) for a review). As a consequence, little is known regarding gender differences in overall experienced well-being—especially in the developing world—and our paper therefore attempts to bring new evidence to this literature. Specifically, we explore the roles of time use and activity-specific affective experiences for these gender differences in experienced well-being using data on older adults from five developing countries. Our analysis exploits detailed data from an abbreviated version of the Day Reconstruction Method (DRM) that was administered in the first—and currently only available—wave (2007–2010) of the World Health Organization’s (WHO) Study on Global Aging and Adult Health (SAGE). The inclusion of DRM data in SAGE offers a unique opportunity to deconstruct experienced well-being into the two components already put forward in the context of Edgeworth’s hedinometer: time use and affective experiences during each activity. To the best of our knowledge, no other survey provides harmonized DRM data in multiple countries, especially no aging survey in low- or middle-income countries. While we do not aim to conduct detailed cross-country comparisons in this paper, we use the multiple analyses of data from different study sites as an opportunity to validate our findings across different cultural and geographic settings from around the world.

To further deconstruct any gender differences in experienced well-being, we separately analyze potential gender differences in time-use on the one hand, and activity-specific net affects on the other. These analyses allow us to evaluate the relative importance of each of the two constituent parts of experienced well-being. We then isolate the relative contributions of gender differences in time-use (“*time composition effects”*) and gender differences in activity-specific affective experiences (“*saddening effects”*), by comparing hypothetical levels of experienced well-being based on counterfactual thought experiments which eliminate existing gender differences in activity-specific net affects and gender differences in time use, respectively. These thought experiments are analogous to those reported in Flores et al. (2015) for analyzing differences in experienced well-being between older adults with and without disabilities, and are similar in spirit to analyses by Knabe et al. (2010), who compare the experienced well-being of employed and unemployed individuals in Germany.

Our study contributes to the existing evidence in several ways. Building on previous research on gender differences in various dimensions of subjective well-being among older adults in low- and middle-income countries (Kieny et al. 2020b), we provide a detailed analysis of gender differences in experienced well-being that isolates the relative roles of gender differences in time use vs. gender differences in activity-specific affective experiences to account for any differences in experienced well-being between men and women. Discussions on gender differences in subjective well-being in academia and policy often highlight gender differences in time use and “time poverty” (e.g. Wodon and Blackden 2006; Walker 2013; Sweet and Kanaroglou 2016), especially related to the generally female double burden of performing both professional and house work. Furthermore, we use two different statistical models to assess any gender differences in experienced well-being and deconstruct these differences into corresponding gender differences in time use and activity-specific net affects. Specifically, we first perform our analyses adjusting first for age only (*age*-*adjusted* models) before moving to richer statistical models that also adjust for a larger set of covariates related to respondents’ socio-demographic characteristics, health status and community environment beyond age (*fully*-*adjusted* models). Comparing and contrasting gender differences based on *age*-*adjusted* and *fully*-*adjusted* models allows us to first assess age-adjusted sub-population differences in experienced well-being between men and women along with their sources in terms of corresponding gender differences in time use and activity-specific net affects, which may be most relevant for an overall assessment of gender inequalities in experienced well-being. Moving to the *fully*-*adjusted* models, in turn, allows us to be more specific about the potential roles of gender per se vs. gender differences in general life circumstances for resulting gender differences in experienced well-being, time use and activity-specific net affects. These analyses may provide important insights on the mechanisms underlying these gender inequalities and suggest avenues for potential policy levers and interventions aimed at closing the subjective well-being gap. Our approach is thereby inspired by mediation-type analyses commonly used to explore different mechanisms linking a specific outcome of interest (here: experienced well-being) with an independent variable of special interest (here: gender) and allows us to side-step the long-standing debate regarding the use of control variables in research on subjective well-being by performing our analyses in both ways, i.e., without and with a comprehensive set of control variables (Blanchflower and Oswald 2008; Glenn 2009; Blanchflower and Oswald 2009).

## Data and Measures

### Data

We use data from the first wave of the World Health Organization’s Study on Global Ageing and Adult Health (SAGE), collected between 2007 and 2010. SAGE is an internationally harmonized survey on aging in low- and middle-income settings, whose data collection activities are mostly focused on adults aged 50 and older. As SAGE only collected data from relatively small comparison samples of younger adults aged 18 to 49 years old, we focus our analysis on the relationship between gender and experienced well-being among adults aged 50 and older, which represented the main target population of SAGE. While SAGE data is collected in six low-and middle-income countries—China, Ghana, India, Mexico, the Russian Federation and South Africa—we exclude Mexico (2070 observations) from our analysis because close to 50 percent of the Mexican sample has missing information on our outcomes of interest from the well-being module due to incomplete interviews. Using SAGE data enables us to perform parallel analyses for countries in different regions of the world and across different cultural contexts based on fully harmonized data. Such parallel analyses allow us to determine whether our results are robust across multiple settings and, therefore, whether they represent a general pattern rather than some country-specific idiosyncratic associations due to specific cultural contexts or location.

SAGE contains individual- and household-level data, including information about respondents’ socio-demographic characteristics, their social environment, health and healthcare use, and well-being. A key asset of SAGE consists in the inclusion of comprehensive assessments of emotional well-being. Notably, the administration of an abbreviated DRM instrument to measure experienced well-being generates a combination of time-use data with corresponding reports of individuals’ affective experiences during the reported activities. In the abbreviated DRM instrument of SAGE, individuals are randomly allocated into one of four groups, which are in turn asked to report on their time use and affective experiences over the course of the previous morning, afternoon, evening or entire day, respectively. We drop from our sample the 7649 individuals that were randomly assigned to the full-day group, as those respondents do not report the detailed time diary data and corresponding activity-specific affects needed to construct our measure of experienced well-being. Finally, we eliminate 1660 additional observations with missing information on any of the covariates used in the analysis. Following the above sample selection procedures, our final sample consists of 21,488 respondents, including 9106 observations from China, 3031 from Ghana, 4833 from India, 2513 from Russia, and 2005 from South Africa.

### Experienced Well-Being

We use experienced well-being as a measure of emotional well-being. In order to construct this measure, we combine the data on time use and activity-specific affects provided by SAGE’s abbreviated DRM instrument.^3^ Individuals from each of the three randomly assigned DRM groups—the “morning”, “afternoon”, and “evening” groups—are asked to report their time use during their respective parts of the previous day, i.e., starting after waking up for the morning group, at noon for the afternoon group or at 6 pm for the evening group. Respondents report what they have been doing based on a list of 22 different activities. They then report how much time they spent on each specific activity and with whom they interacted during the activity. The abbreviated DRM module thereby elicits information for up to ten successive activities or until the interview time reaches 15 min for the DRM section. For each activity, respondents provide further information on the prevalence and intensity of two positive emotions (feeling calm or relaxed, and feeling enjoyment), and five negative emotions (feeling worried, rushed, irritated or angry, depressed, tense or stressed). The intensity of each positive and negative affect during an activity is measured on a three-point scale. We aggregate these reported intensities of positive and negative affective experiences into a single measure of “net affect”. We simultaneously use the data from all three DRM groups in our estimations in order to ensure that our estimates of time-use and corresponding affective experiences represent those of the entire day in the target population of individuals aged 50 years and older (Miret et al. 2012).

The large number of activities included in the activity list implies that some of the 22 activities are reported with rather low frequencies. To address the issue of infrequent activities and to facilitate statistical estimation, we follow previous research (Flores et al. 2015; Kieny et al. 2020a, b) and aggregate the 22 activities into five broader activity groups^4^: work, housework, travel, leisure, and self-care. This reclassification of activities aims at striking a balance between grouping activities into relatively intuitive and easily interpretable categories while avoiding small prevalence rates for very specific and infrequent activity groups that would be challenging to integrate into our econometric framework. Note that we refer to “work” exclusively as paid work and subsistence farming, excluding any (unpaid) household-related tasks, which are part of the separate category “housework”.

We define experienced well-being based on Kahneman’s definition as the “integral of the stream of pleasures and pains associated with events over time” (Kahneman et al. 2004). Formally, experienced well-being can be represented as the duration-weighted sum of net affects for all activities performed during the day, that is:

1 $$U_{i} = \mathop \sum \limits_{a} \tau_{ia} u_{ia}$$

where $u_{i,a}$ represents individual $i$’s net affect during activity $a$, and $\tau_{ia} = \frac{{T_{ia} }}{{T_{i} }}$ represents the share of non-sleeping time that individual $i$ spends on activity $a$, that is: $T_{ia}$, the duration of activity a, over $T_{i}$, the total time covered by the up to 10 successive activities that the respondent reported during her assigned time window.

Following Kahneman and Krueger’s definition of net affect (Kahneman and Krueger 2006), individual $i$’s net affect during activity $a$, is defined as:

2 $$u_{ia} = \mathop \sum \limits_{\text{s}} \left( {\mathop \sum \limits_{\text{l}} h_{is} PA_{is}^{l} - \mathop \sum \limits_{\text{k}} h_{is} NA_{is}^{k} } \right) \forall a = 1, \ldots , 5$$

where $PA_{is}^{l}$ is the $l$’th positive affect and $NA_{is}^{k}$ is the $k$’th negative affect that person $i$ reports for each spell $s$ of possibly multiple reports of activity $a$. We control for multiple occurrences of the same activity by taking the time-weighted average of positive and negative affect scores. The weight $h_{is}$ is defined as:

3 $$h_{is} = \frac{{t_{is} }}{{T_{ia} }}$$

where $t_{is}$ is the duration of one specific occurrence of activity $a$ and $T_{ia}$ is the total time spent on activity $a$ during the assigned period of time. In other words, the net affect of activity $a$ is the weighted sum of positive and negative affects experienced during different occurrences of activity $a$ over the assigned time period, whereby the weights correspond to the relative time share of each specific occurrence of activity $a$ relative to the total time spent on activity $a$ during the assigned reporting period.

To be able to more clearly highlight activities that generally result in above or below average affective experiences and to facilitate the comparative interpretation of our estimated coefficients across countries as multiples of the standard deviations of the country-specific distribution of unstandardized experienced well-being, we standardize our measure of activity-specific net affect as follows:

4 $$\tilde{u}_{ia} = \frac{{u_{ia} - \mu_{U} }}{{\sigma_{U} }} \forall a = 1, \ldots , 5$$

where $\mu_{U}$ represents mean and $\sigma_{U}$ the standard deviation of the country-specific distributions of $u_{ia}$. $\tilde{u}_{ia}$, therefore, represents the utility that individual $i$ derives from activity $a$ over the randomly assigned time period, relative to the overall experienced well-being of all individuals across all activities in that country. This standardization allows a more straightforward interpretation, as the sign of $\tilde{u}_{ia}$ indicates whether the net affect associated with activity $a$ is above or below the mean net affect across all activities in the respective country under consideration.

Finally, we construct a standardized version of the overall experienced well-being as follows:

5 $$\tilde{U}_{i} = \frac{{\mathop \sum \nolimits_{a} \tau_{ia} u_{ia} - \mu_{U} }}{{\sigma_{U} }}$$

which measures the average experienced well-being of individual $i$ over her assigned time period, standardized based on the overall distribution of experienced well-being of all individuals from the same country. This standardized measure represents the main outcome of interest for our overall analyses of gender differences in experienced well-being. This final standardization ensures that our estimates of the gender (and other) coefficients can be interpreted in standard deviation units, i.e., as relative to the country-specific distribution of unstandardized experienced well-being, which may enhance the comparability of our estimates across countries, especially if we suspect the unstandardized distributions of experienced well-being across countries to be different for reasons that are unrelated to actual experienced well-being, such as issues of survey design or country-specific response scales.

### Explanatory Variables

The *age*-*adjusted* analysis controls only for age in addition to gender, while the *fully*-*adjusted* analysis also includes control variables related to respondents’ sociodemographic and economic status, as well as health status and social cohesion, which could be correlated with both gender and experienced well-being. The inclusion of control variables allows us to identify and quantify potential channels underlying any potential subpopulation differences in experienced well-being between men and women. The sociodemographic and economic control variables include age, marital status, household composition (number of adults and children living in the household), whether respondents live in an urban or rural area, years of education and employment status. These variables are significantly correlated with gender in at least a subset of study countries (Table 1), and we hypothesize that they may also influence experienced well-being and thus represent potential mechanisms or confounders in the relationship between gender and experienced well-being. Although we cannot control directly for individual resources, we use household income quartiles based on SAGE’s permanent income variable as a proxy for living standards of individual household members. Moreover, in order to account for potential differences in the within-household income distribution, we also include an individual-level explanatory variable indicating whether respondents report having enough money to meet their own needs. The health variables include a WHO disability index and a measure of self-assessed pain. Specifically, we use the 12-item version of the WHO Disability Assessment Schedule (WHODAS 2.0), an index which captures different aspects of disability, following the definition of the International Classification of Functioning, Disability and Health (World Health Organization 2001). The WHODAS 2.0 concentrates on six life domains: cognition, mobility, self-care, getting along, life activities, and participation. Self-assessed pain measures the degree of pain or bodily discomfort that the respondent reported experiencing during the previous month, and whether this pain induced difficulties in everyday life. Finally, we use community involvement, trust in others, perceived safety in the neighborhood and having been a victim of a violent crime in the last 12 months as measures of social cohesion. Community involvement measures the level of participation to social activities, while trust in others measures how much the individual has confidence in different groups of people, such as co-workers, neighbors or strangers.

## Econometric Models and Counterfactual Analysis

### Experienced Well-Being

To assess whether there is an age-adjusted gender gap in experienced well-being, we first regress our standardized measure of experienced well-being $\tilde{U}_{i}$ on a dummy for gender, only including age as an additional control variable. We thus estimate the *age*-*adjusted* overall experienced well-being gap as follows:

6 $$\tilde{U}_{i} = \alpha + \beta^{A} Female_{i} + {{\vartheta }}^{A} Age_{i} + \varepsilon_{i }$$

In a second step, we explore how the partial association between gender and overall experienced well-being changes once we control for additional measures of individuals’ life circumstances. We, therefore, perform the same regression, but this time including an expanded set of control variables into the model $X_{i}$, estimating the *fully*-*adjusted* gender gap in experienced well-being as follows:

7 $$\tilde{U}_{i} = \alpha + \beta^{F} Female_{i} + X_{i} \gamma^{F} + \varepsilon_{i}$$

We estimate these two regressions using OLS, adding sample weights, to ensure the correct estimation of the corresponding conditional means of experienced well-being across population groups (Solon et al. 2013).

In order to further deconstruct any gender differences in experienced well-being, we also analyze the two components of experienced well-being—time use and activity-specific net affect—separately.

### Time Use

We estimate the partial association between gender and time use using weighted multivariate fractional logit models, which impose that the estimated time shares fall in the 0–1 interval ($\tau_{ia} \in \left[ {0,1} \right]$), as well as sum up to 1 ($\mathop \sum \nolimits_{a = 1}^{5} \tau_{ia} = 1$). We start by evaluating whether there are any differences in the way men and women spend their time adjusting for age alone (*age*-*adjusted* models). Following Mullahy (2015), we use a multinomial logit functional form such that:

8 $$\xi \left[ {\tau_{a} |X_{i} } \right] = \frac{{\exp \left( {\alpha_{a}^{\tau ,A} + \beta_{a}^{\tau ,A} Female_{i} + \theta_{a}^{\tau ,A} Age_{i} } \right)}}{{1 + \mathop \sum \nolimits_{m = 1}^{4} \exp \left( {\alpha_{m}^{\tau ,A} + \beta_{m}^{\tau ,A} Female_{i} + \theta_{m}^{\tau ,A} Age_{i} } \right)}}\forall a = 1, \ldots , 4$$

9 $$\xi \left[ {\tau_{5} |X_{i} } \right] = \frac{1}{{1 + \mathop \sum \nolimits_{m = 1}^{4} { \exp }\left( {\alpha_{m}^{\tau ,A} + \beta_{m}^{\tau ,A} Female_{i} + \theta_{m}^{\tau ,A} Age_{i} } \right)}}$$

where we impose $\alpha_{5}^{\tau }$ = $\beta_{5}^{\tau }$ = $\theta_{5}^{\tau } = 0$ as a normalization for identification (Cameron and Trivedi 2005).

We then repeat this analysis, this time including the whole set of control variables into the model to assess how men and women’s time use would differ if they had otherwise comparable life circumstances.

10 $$\xi \left[ {\tau_{a} |X_{i} } \right] = \frac{{\exp \left( {\alpha_{a}^{\tau ,F} + \beta_{a}^{\tau ,F} Female_{i} + X_{i} \gamma_{a}^{\tau ,F} } \right)}}{{1 + \mathop \sum \nolimits_{m = 1}^{4} \exp \left( {\alpha_{m}^{\tau ,F} + \beta_{m}^{\tau ,F} Female_{i} + X_{i} \gamma_{m}^{\tau ,F} } \right)}}\forall a = 1, \ldots , 4$$

11 $$\xi \left[ {\tau_{5} |X_{i} } \right] = \frac{1}{{1 + \mathop \sum \nolimits_{m = 1}^{4} { \exp }\left( {\alpha_{m}^{\tau ,F} + \beta_{m}^{\tau ,F} Female_{i} + X_{i} \gamma_{m}^{\tau ,F} } \right)}}$$

We estimate the above equations using quasi-maximum likelihood. However, the empirical distribution of a vector of shares conditional on a set of control variables may suffer from underdispersion (Mullahy 2015). Consequently, the quasi-maximum likelihood procedure may not yield consistent estimates of the covariance matrix. To address these issues, we use a bootstrapping procedure with 250 repetitions to estimate our standard errors.

### Activity-Specific Net Affect

In order to assess whether men and women experience activities differently, we first estimate the *age*-*adjusted* partial associations between gender and activity-specific net affect. We use a weighted linear regression of the form:

12 $$\tilde{u}_{ia} = \alpha_{a}^{A} + \beta_{a}^{A} Female_{i} + \vartheta_{a}^{A} Age_{i} + \epsilon_{ia}^{A} \forall a = 1, \ldots ,5$$

based on the sample that reported activity $a$, using sample weights as described earlier.

We then evaluate gender differences in activity-specific affective experiences, conditional on life circumstances in a similar fashion within the context of *fully*-*adjusted* model that incorporates our expanded set of covariates $X$, that is:

13 $$\tilde{u}_{ia} = \alpha_{a}^{F} + \beta_{a}^{F} Female_{i} + X_{i} \gamma_{a}^{F} + \epsilon_{ia}^{F} \forall a = 1, \ldots , 5$$

It is worth emphasizing that we do not account for potential selection into activities, which does not allow for causal interpretation.

### Time Use vs. Activity-Specific Affective Experiences

Finally, we combine the results from the two separate analyses of gender differences in time use and gender differences in affective experiences in order to assess the relative importance of these differences in time use vs. activity-specific affective experiences to account for the overall gender differences in experienced well-being. Our thought experiments for deconstructing the gender differences in experienced well-being are similar to those of Flores et al. (2015) who assessed the role of disability for experienced well-being. Like in the case of the above regression analyses, we perform these counterfactual thought experiments twice, once controlling only for age, and a second time using our full set of control variables. These analyses help in deconstructing the raw gender differences in experienced well-being (adjusting for age only) as well as to assess the relative contributions of gender differences in time use vs. activity-specific net affects for gender differences in experienced well-being once other differences in life circumstances are also accounted for.

To isolate the contribution of differences in time use, we estimate a so-called *time composition effect* as:

14 $$\Delta_{U}^{Time} = \mathop \sum \limits_{a} \overline{{\tilde{u}}}_{a} \times \delta_{a}^{\tau }$$

where $\overline{{\tilde{u}}}_{a}$ represents the average net affect during activity $a$ and $\delta_{a}^{\tau } = \frac{{\partial \tau_{a} }}{\partial Female}$ are the partial effects of *female* on the proportion of time spent in activity $a$, as calculated in Eqs. (8) and (9) (for the *age*-*adjusted* model) or (10) and (11) (for the *fully*-*adjusted* model). The *time composition effect* describes how men and women’s experienced well-being would differ if both genders had the same activity-specific affective experiences (activity-specific net affect being set at the overall country-average, irrespective of gender), but their time use would continue to differ by gender. In other words, would men or women have higher experienced well-being, if everyone would experience all activities in the same way, but gender differences in time use would remain as observed in the data?

To isolate the contribution of gender differences in affective experiences, we estimate the so-called *saddening effect* as:

15 $$\Delta_{U}^{Affect} = \mathop \sum \limits_{a} \bar{\tau }_{a} \times \delta_{a}^{{\tilde{u}}}$$

where $\delta_{a}^{{\tilde{u}}} = \frac{{\partial \tilde{u}_{a} }}{\partial Female}$ represent the partial effects of female on activity-specific net affect of activity $a$, as calculated in Eqs. (12) and (13), for the *age*-*adjusted* and *fully*-*adjusted* regressions, respectively. The *saddening effect* describes how men and women’s experienced well-being would differ if both genders were not to differ in their activity patterns (time use being set at the overall country-average, irrespective of gender), but their activity-specific net affect were to remain gender-specific. In other words, would men or women have higher experienced well-being, should both genders spend their day in exactly the same way, while still having gender-specific affective experiences associated with these activities?

Although our analysis is broadly comparable to the decomposition analysis performed by Knabe et al. (2010) to study well-being differences between employed and unemployed individuals in Germany, our approach differs in two important ways. Firstly, their estimations of the *saddening* and *time composition effects* are based on unconditional group differences, while we control either for age alone or for a large set of control variables. Secondly, Knabe et al. (2010) define the *time composition effect* as a residual effect obtained by subtracting the *saddening effect* from the overall differences in experienced well-being between the two groups under consideration, while we define the two effects symmetrically, even if this implies that the two effects do not add up to the overall group differences in experienced well-being due to an omitted interaction term.

Finally, while our deconstruction of the gender differences in experienced well-being bears some similarities with other econometric decompositions techniques, such as the Oaxaca–Blinder decomposition, our aim is fundamentally different. Oaxaca-Blinder decompositions generally examine how unconditional mean differences in an outcome across groups may be attributed to group differences in explanatory variables on the one hand and their group-specific associations with the outcome of interest on the other. By contrast, our analyses aim to isolate and quantify the respective contributions of each constituent part of experienced well-being—time use and activity-specific net affect—for differences in the overall experienced well-being of older men and women. We, therefore, need to apply alternative techniques to construct meaningful counterfactuals for obtaining our *saddening* and *time composition effects*, as outlined in Flores et al. (2015).

## Results

### Descriptive Statistics

Table 1 presents key characteristics of our analytical sample, i.e., the country- and gender-specific averages of all explanatory variables used in our analyses.

**Table 1:** Life circumstances of men and women

	Ghana		India		China		South Africa		Russia	
	Men	Women	Men	Women	Men	Women	Men	Women	Men	Women
*Demographics*										
Age	64.16	64.27	61.45	61.40	62.04	**62.90**	60.88	61.76	61.75	**64.87**
Age 50–59	0.42	0.39	0.50	0.47	0.47	**0.44**	0.53	0.49	0.56	**0.42**
Age 60–69	0.27	0.28	0.30	0.32	0.32	0.31	0.31	0.31	0.22	0.24
Age 70–79	0.23	0.24	0.16	0.16	0.17	**0.21**	0.11	**0.16**	0.15	**0.25**
Age 80+	0.09	0.09	0.04	0.05	0.04	**0.05**	0.05	0.05	0.07	**0.10**
Married	0.85	**0.23**	0.91	**0.61**	0.91	**0.80**	0.72	**0.32**	0.71	**0.41**
Rural	0.60	0.58	0.75	0.73	0.57	**0.50**	0.37	0.39	0.31	0.26
Number of adults in household	3.86	**3.31**	4.98	4.69	2.50	**2.44**	3.00	3.01	2.42	**2.06**
Number of children in household	2.01	**1.79**	1.88	1.97	0.21	0.22	0.78	**0.98**	0.17	0.15
*Socioeconomic Status*										
Working	0.73	**0.67**	0.64	**0.21**	0.53	**0.35**	0.45	**0.25**	0.50	**0.35**
Less than primary	0.53	**0.76**	0.42	**0.82**	0.31	**0.54**	0.45	**0.51**	0.01	**0.03**
Primary completed	0.13	**0.10**	0.19	**0.10**	0.25	**0.17**	0.24	0.21	0.05	0.05
Secondary	0.06	**0.02**	0.15	**0.04**	0.23	**0.16**	0.12	0.15	0.16	0.23
Highschool	0.24	**0.10**	0.15	**0.03**	0.15	**0.10**	0.12	0.09	0.59	0.52
College or higher	0.05	**0.02**	0.09	**0.02**	0.06	**0.03**	0.07	**0.03**	0.19	0.18
*Income*										
Q1: permanent income	0.21	**0.25**	0.23	0.24	0.21	0.21	0.29	0.25	0.18	**0.23**
Q2: permanent income	0.23	**0.28**	0.23	0.24	0.23	0.24	0.24	**0.31**	0.21	**0.27**
Q3: permanent income	0.26	0.26	0.25	0.23	0.30	0.28	0.19	0.23	0.24	0.24
Q4: permanent income	0.30	**0.21**	0.29	0.28	0.27	0.27	0.28	**0.22**	0.38	0.25
Enough money to meet one’s needs	0.33	**0.26**	0.68	**0.63**	0.80	0.79	0.38	**0.33**	0.75	0.74
*Health*										
WHO disability score	2.33	**2.65**	2.50	**2.93**	2.89	**3.07**	2.18	**2.42**	2.38	**2.73**
Self-assessed pain	1.36	**1.61**	1.10	**1.47**	0.78	**0.97**	1.01	**1.19**	0.83	**1.18**
*Social Environment*										
Trust	3.21	**3.07**	3.40	**3.01**	5.84	5.81	2.76	2.80	3.02	3.02
Safety	4.94	**4.79**	3.72	**3.52**	5.57	**5.24**	2.39	**2.23**	3.13	2.84
Community involvement	3.60	**3.32**	3.81	**3.08**	4.34	**4.23**	3.99	**3.82**	3.87	3.84
Victim of a violent crime (last 12 m)	0.04	0.04	0.03	0.03	0.01	0.02	0.08	**0.05**	0.01	0.02
Number of observations	1577	1454	2441	2392	4288	4818	793	1212	950	1690

The entries in each column are country-specific averages using population weights. The average under Women is bold whenever there is a significant difference between genders in a pairwise comparison (*p* < 0.10). Permanent income quartiles are country-specific and derived from an asset index. Trust is a score based on questions about perceived trust in neighbors, colleagues and strangers. Safety is a score based on information about perceived safety in the neighborhood and whether the respondent has been a victim of a violent crime. Community involvement measures the degree of participation in social activities such as attending clubs or public meetings, or socializing with co-workers

As highlighted in the table, men and women in our sample have substantially different characteristics and life circumstances. In all countries, women are less likely to be married than men, a likely reflection of gender differences in life expectancy and a correspondingly higher prevalence of widowhood among women than men. Women also tend to live in smaller households in all but one country (South Africa). In addition, women work less often than men, with a 6–43 percentage points difference across the five countries. Except for Russia, where a majority of individuals have relatively high levels of education, irrespective of gender, women generally report a substantially lower education level than men. In particular, a much larger share of women than men have not completed primary school education. This gap is as high as a 40 and 23 percentage points for India and Ghana/China, respectively. Women also tend to live in poorer households than men. Furthermore, in Ghana, India and South Africa, women are significantly less likely than men to report having enough money to meet their personal needs. In all countries, women report a higher level of self-assessed pain and suffer from higher levels of disability, which highlights poorer health status among older women than men. Finally, women generally report feeling less safe and tend to be less often involved in community activities.

Table 2 shows country- and gender-specific descriptive statistics of standardized experienced well-being, time use and activity-specific net affect.

**Table 2:** Descriptive statistics of men and women

	Ghana		India		China		South Africa		Russia	
	Men	Women	Men	Women	Men	Women	Men	Women	Men	Women
*Panel A. Experienced well-being*	0.048	**− 0.052**	0.075	**− 0.078**	0.022	**− 0.022**	0.083	**− 0.052**	0.110	**− 0.076**
*Panel B. Time use*										
Working	0.212	**0.178**	0.191	**0.057**	0.218	**0.127**	0.175	**0.085**	0.298	**0.189**
Housework	0.072	**0.181**	0.099	**0.297**	0.115	**0.279**	0.109	**0.234**	0.116	**0.328**
Travel	0.075	**0.049**	0.084	**0.043**	0.033	**0.025**	0.061	**0.041**	0.108	0.050
Leisure	0.472	**0.428**	0.439	0.427	0.514	**0.456**	0.465	0.427	0.364	0.331
Self-care	0.164	0.159	0.187	0.177	0.12	**0.113**	0.179	0.197	0.114	0.103
*Panel C. Activity*-*specific net affect*										
Working	**− **0.220	− 0.324	− 0.295	− 0.324	− 0.352	− 0.315	− 0.126	− 0.303	− 0.397	− 0.419
Housework	− 0.054	− 0.064	0.004	**− 0.331**	− 0.107	**− 0.165**	0.117	**− 0.047**	0.116	**− 0.128**
Travel	− 0.132	− 0.232	0.004	**− 0.299**	0.014	− 0.01	0.043	− 0.130	0.202	**− 0.335**
Leisure	0.241	**0.131**	0.208	**0.048**	0.198	0.189	0.235	**0.011**	0.376	0.265
Self-care	0.211	0.238	0.264	0.138	0.152	0.134	0.152	0.111	0.582	**0.318**
Number of observations	1577	1454	2441	2392	4288	4818	793	1212	950	1690

The entries in each column are country-specific averages using population weights. The average under Women is bold whenever there is a significant difference between genders in a pairwise comparison (*p* < 0.10). Time Composition averages represent the share of time spent in activity *a* over the reported period, while Activity-Specific Net Affects are calculated as in Eq. (4)

Panel A presents weighted averages of standardized experienced well-being for men and women in each country. While the overall weighted average of standardized experienced well-being across both genders is zero by construction, experienced well-being is significantly lower for women than for men in all countries. Since experienced well-being is standardized at country level using population-weighted means and standard deviations of country-specific distribution, the absolute magnitude of differences cannot be compared between countries. Panel B presents the unadjusted country- and gender-specific average time shares spent on each activity group. We observe the usual patterns of traditional gender roles. In addition, even when adding up the time spent working and traveling, the overall amount of time spent on work and housework combined is larger among women than men. Panel C shows country- and gender-specific estimates of activity-specific net affects for all activity groups. For both genders, the three activities associated with the worst affective experiences in all countries are work, travel and housework. In addition, work is nearly always rated as the activity leading to the lowest levels of net affect. While housework, work and travel yield strictly negative (i.e., below average) affective experiences for women in all countries, the situation is more nuanced for men. Indeed, men tend to have below average affective experiences when working, but this is not always the case for housework or travel. Self-care and leisure are always associated with positive (i.e., above average) net affect. If we consider work, travel and housework as part of a wider category of work-related activities, and self-care and leisure as part of more leisurely activities, the ranking of activities in all countries is consistent with a neoclassical utility function that assumes that individuals prefer leisure over work. Finally, the pairwise comparisons of net affect by gender show that, while there are no significant differences in terms of how much men and women “dislike” work, women have significantly worse affective experiences doing housework than men in all but one country. Compared to men, women also report significantly lower levels of net affect associated with leisure in three out of five countries.

### Analysis

Table 3 presents country-specific population-weighted estimates of the partial associations of gender and experienced well-being in both the *age*-*adjusted* and *fully*-*adjusted* models.

**Table 3:** Partial association of gender with experienced well-being *U* for individuals aged 50+

	Ghana	India	China	South Africa	Russia
Age-adjusted difference	− 0.102**	− 0.154***	− 0.049**	− 0.142*	− 0.195**
Fully-adjusted difference	− 0.054	− 0.082	0.007	− 0.033	− 0.106

Standard errors are computed using 250 bootstrap replications. Sample weights are applied. The entries in each column are country-specific average partial effects of gender on experienced well-being. Average partial effects are based on a linear regression (Eq. (6) for the age-adjusted difference and Eq. (7) for the fully-adjusted). The age-adjusted difference controls only for age, while the fully-adjusted difference controls for a large set of control variables included in Table 1

*p < 0.10, **p < 0.05, ***p < 0.01

When controlling for age only, women are at a significant disadvantage compared to men in all countries, with corresponding gender gaps in experienced well-being ranging from 0.05 standard deviations in China to 0.2 standard deviations in Russia. However, when incorporating the larger set of individual characteristics and life circumstances into our model, any remaining gender differences in experienced well-being become statistically insignificant in spite of remaining negative in all countries but China.

Looking at the coefficients of our control variables (Appendix 1), we observe that the most important factors associated with experienced well-being are disability and access to income (especially being part of the top quartile of the household income distribution). We therefore hypothesize that the experienced well-being gap observed between men and women is mostly due to women’s individual characteristics and life circumstances, and in particular to the fact that their health status is often worse than that of men (higher WHO disability score) and that they generally live in poorer households.

Table 4 presents population-weighted estimates of the partial associations between gender and time use, based on country-specific multivariate fractional logit models. By construction, all country-specific partial associations must sum up to zero as the activities considered are both exhaustive and mutually exclusive.

**Table 4:** Partial association of gender with time shares τ_a_ for individuals aged 50+

	Ghana	India	China	South Africa	Russia
*Panel A. Age*-*adjusted differences in time use*					
Work	**− **0.032***	**− **0.135***	**− **0.0835***	**− **0.080***	**− **0.072**
Housework	0.111***	0.197***	0.1673***	0.1345***	0.213***
Travel	**− **0.025***	**− **0.039***	**− **0.0081***	**− **0.0188***	**− **0.048
Leisure	**− **0.047***	**− **0.013	**− **0.0671***	**− **0.0515**	**− **0.076***
Self-care	**− **0.006	**− **0.009	**− **0.0086***	0.0158	**− **0.016
*Panel B. Fully*-*adjusted differences in time use*					
Work	**− **0.032**	**− **0.055***	**− **0.051***	**− **0.056***	**− **0.043
Housework	0.116***	0.219***	0.159***	0.120***	0.217***
Travel	**− **0.020***	**− **0.027***	**− **0.009***	**− **0.014**	**− **0.039*
Leisure	**− **0.070***	**− **0.113***	**− **0.089***	**− **0.062***	**− **0.107***
Self-care	0.006	**− **0.024***	**− **0.010***	0.011	**− **0.028

Standard errors are computed using 250 bootstrap replications. Sample weights are applied. The entries in each column are country-specific average partial effects of gender on time shares. Average partial effects are based on a multivariate fractional logit model (Panel A. Equations (8) and (9) and Panel B. Equations (10) and (11)). Panel A. controls only for age, while Panel B. controls for a large set of control variables included in Table 1

*p < 0.10, **p < 0.05, ***p < 0.01

Panel A shows the results from models that control for age only while Panel B refers to models that control for a wide range of individual characteristics and life circumstances (see Appendix 2 for the detailed coefficients of the covariates). The results show a pattern that is roughly comparable to the descriptive statistics presented above: women spend significantly less time than men on work and travel, and more time on housework. This finding is consistent across all countries, and changes relatively little when we add additional controls for individual characteristics and life circumstances. In addition, similar to findings reported in the 2012 World Development Report (World Bank 2012), our analysis reveals a much smaller gender gap in time spent working than in time spent doing housework, which indicates that women tend to spend more time on work and housework combined and less time on leisure activities compared to men.

Table 5 presents country-specific population-weighted estimates of the partial associations between gender and activity-specific net affects, controlling first for age only (Panel A) and then for our entire set of individual control variables (Panel B).

**Table 5:** Partial association of gender with activity-specific net affects u_a_

	Ghana	India	China	South Africa	Russia
*Panel A. Age*-*adjusted differences in activity*-*specific net affects*					
Work	**− **0.104	**− **0.041	0.027	**− **0.157	**− **0.023
Housework	**− **0.014	**− **0.321***	**− **0.059*	**− **0.155**	**− **0.235**
Travel	**− **0.098	**− **0.305***	**− **0.045	**− **0.17	**− **0.414
Leisure	**− **0.112**	**− **0.159***	**− **0.009	**− **0.232***	**− **0.114
Self-care	0.027	**− **0.126***	**− **0.0212	**− **0.046	**− **0.212**
*Panel B. Fully*-*adjusted differences in activity*-*specific net affects*					
Work	**− **0.076	0.099	0.109**	0.187	0.0187
Housework	0.046	**− **0.113	0.011	**− **0.070	**− **0.152
Travel	**− **0.029	**− **0.240**	0.004	0.031	**− **0.240
Leisure	**− **0.078	**− **0.062	0.048**	**− **0.128**	0.043
Self-care	0.034	**− **0.019	0.047**	0.053	**− **0.125

Standard errors are computed using 250 bootstrap replications. Sample weights are applied. The entries in each column are country-specific average partial effects of gender on Activity-Specific Net Affects. Average partial effects are based on a linear regression (Panel A. Equation (12) and Panel B. Equation (13)). Panel A. controls only for age, while Panel B. controls for a large set of control variables included in Table 1

*p < 0.10, **p < 0.05, ***p < 0.01

All but two of the estimated coefficients in Panel A are negative, although many are not statistically different from zero at conventional levels of significance. In all countries but Ghana, women report significantly worse net affects associated with housework than men, with corresponding differences ranging from about 0.06 standard deviations in China to about 0.32 in India. In addition, in three out of five countries, women also report significantly worse levels of net affect during leisure activities than men. In general, when controlling for age only, it appears that women report worse net affects for all activities, even if the difference is not always statistically significant. Controlling for additional individual characteristics and life circumstances (Panel B) reduces the statistical significance of any gender differences in activity-specific net affect even further. More importantly though, many of the estimated coefficients change their sign in the *fully*-*adjusted* models. In China, all the estimated partial associations between being a woman and the activity-specific net affects are positive in the *fully*-*adjusted* models, and these associations are also statistically significant in the cases of work, leisure and self-care. By contrast, no clear pattern for gender differences in activity-specific net affects emerges across the other countries once additional control variables are incorporated into the models. The fact that incorporating further controls for individual characteristics and life circumstances into our model substantially attenuates the association between gender and activity-specific net affect suggests that other factors like health status and economic position may be able to largely explain the worse activity-specific affective experiences of women compared to men (Appendix 3).

We now combine the results from the above analyses within the framework of a hypothetical thought experiment aimed at assessing the relative importance of gender differences in time use (*time composition effect*) and activity-specific net affects (*saddening effect*) for gender differences in experienced well-being. Like in our earlier analyses, we first incorporate only age as a control variable before including a full set of controls for individual characteristics and life circumstances into our estimations. Table 6 first presents the overall gender differences in experienced well-being and deconstructs these into two components: *time composition* and *saddening effects*, while Tables 7 and 8 provide the results of further disaggregated analyses at the level of individual activities. The *time composition effect* isolates gender differences in experienced well-being attributable to gender differences in time use by fixing activity-specific net affect at the country-specific averages (irrespective of gender) and computing hypothetical gender differences in experienced well-being if men and women would only differ in terms of their activity patterns. The *saddening effect*, on the other hand, highlights gender differences in experienced well-being attributable to gender differences in activity-specific net affects by fixing time use at the overall country-specific averages (irrespective of gender) and computing hypothetical gender differences in experienced well-being if men and women would only differ in terms of their activity-specific affective experiences.

**Table 6:** Counterfactual partial association of gender with experienced well-being and its time composition and saddening effects for individuals aged 50+

	Ghana	India	China	South Africa	Russia
*Panel A. Age*-*adjusted*					
Difference	**− **0.102**	**− **0.154***	**− **0.049**	**− **0.142*	**− **0.195**
Time composition effect	**− **0.004	**− **0.002	**− **0.010**	0.014	**− **0.015
Saddening effect	**− **0.075*	**− **0.179***	**− **0.015	**− **0.167***	**− **0.155**
*Panel B. Fully*-*adjusted*					
Difference	**− **0.054	**− **0.082	0.007	**− **0.033	**− **0.106
Time composition effect	**− **0.007	**− **0.049***	**− **0.025***	0.008	**− **0.042*
Saddening effect	**− **0.041	**− **0.055	0.050**	**− **0.036	**− **0.049

Standard errors are computed using 250 bootstrap replications. Sample weights are applied. The entries in each column are country-specific differences in standardized experienced well-being between men and women. The time composition effect is computed as in Eq. (14) and the saddening effect is computed as in Eq. (15). Panel A. controls only for age, while Panel B. controls for a large set of variables included in Table 1

*p < 0.10, **p < 0.05, ***p < 0.01

**Table 7:** Counterfactual partial association of gender and its time composition effect (decomposed across activity groups) for individuals aged 50+

	Ghana	India	China	South Africa	Russia
*Panel A. Age*-*adjusted*					
**Time composition effect**	− **0.004**	− **0.002**	− 0.010**	**0.014**	− **0.015**
Work	0.009***	0.041***	0.028***	0.017*	0.030**
Housework	**− **0.007*	**− **0.043***	**− **0.024***	**− **0.000	**− **0.016
Travel	0.004***	0.004*	0.000	0.001	0.002
Leisure	**− **0.009***	**− **0.002	**− **0.01***	**− **0.005	**− **0.024**
Self-care	**− **0.001	**− **0.002	**− **0.001***	0.002	**− **0.007
*Panel B. Fully*-*adjusted*					
**Time composition effect**	− **0.007**	− 0.049***	− 0.025***	**0.008**	− 0.042*
Work	0.009**	0.017***	0.017***	0.012	0.018
Housework	**− **0.007*	**− **0.049***	**− **0.023***	**− **0.000	**− **0.016
Travel	0.004***	0.003*	0.000	0.001	0.002
Leisure	**− **0.013***	**− **0.015***	**− **0.017***	**− **0.006*	**− **0.033***
Self-care	0.001	**− **0.005***	**− **0.002***	0.002	**− **0.012

Standard errors are computed using 250 bootstrap replications. Sample weights are applied. The entries in each column are country-specific differences in standardized experienced well-being between men and women. Each component of the time composition effect is computed as in Eq. (14). Panel A. controls only for age, while Panel B. controls for a large set of variables included in Table 1

*p < 0.10, **p < 0.05, ***p < 0.01

**Table 8:** Counterfactual partial association of gender and its saddening effect (decomposed across activity groups) for individuals aged 50+

	Ghana	India	China	South Africa	Russia
*Panel A. Age*-*adjusted*					
**Saddening effect**	− 0.075*	− 0.179***	− **0.015**	− 0.167***	− 0.155**
Work	**− **0.020	**− **0.005	0.005	**− **0.019	**− **0.005
Housework	**− **0.002	**− **0.063***	**− **0.012*	**− **0.029**	**− **0.057**
Travel	**− **0.006	**− **0.020***	**− **0.001	**− **0.008	**− **0.031
Leisure	**− **0.051**	**− **0.069***	**− **0.004	**− **0.102***	**− **0.039
Self-care	0.004	**− **0.023***	**− **0.003	**− **0.009	**− **0.023**
*Panel B. Fully*-*adjusted*					
**Saddening effect**	− **0.041**	− **0.055**	0.050**	− **0.036**	− **0.049**
Work	**− **0.015	0.012	0.019**	0.022	0.004
Housework	0.006	**− **0.022	0.002	**− **0.013	**− **0.037
Travel	**− **0.002	**− **0.015**	0.000	0.002	**− **0.018
Leisure	**− **0.035	**− **0.027	0.023**	**− **0.057**	0.015
Self-care	0.006	**− **0.004	0.006**	0.010	**− **0.013

Standard errors are computed using 250 bootstrap replications. Sample weights are applied. The entries in each column are country-specific differences in standardized experienced well-being between men and women. Each component of the saddening effect is computed as in Eq. (15). Panel A. controls only for age, while Panel B. controls for a large set of variables included in Table 1

*p < 0.10, **p < 0.05, ***p < 0.01

Panel A of Table 6 shows that, when controlling only for age, gender differences in experienced well-being are mainly driven by the *saddening effects*, that is, by the fact that women have worse affective experiences when performing most activities than men. Indeed, in all countries but China, if both genders had the same (country-specific average) time use patterns, but differed in their activity-specific net affects, women would have statistically significantly lower levels of experienced well-being than men. The corresponding *time composition effects* on the other hand seem relatively small. Panel B shows that when considering additional individual characteristics and life circumstances, gender differences in experienced well-being lose statistical significance. The generally negative—although small—*time composition effects*, on the other hand, become statistically significantly different from zero in three of our study countries. That is, if women and men had exactly the same activity-specific affective experiences (set at the overall country-specific average), the remaining gender differences in time use alone would generally result in lower levels of experienced well-being among women than men. Hence, holding other characteristics and life circumstances fixed, women tend to engage in more unpleasant activities overall than men. Meanwhile, the *saddening effects*—which were negative and statistically significant everywhere but in China in the *age*-*adjusted* models—become insignificant in four countries when we include the whole set of control variables, and even turn significantly positive in China.

Tables 7 and 8 present additional details for this hypothetical thought experiment by showing how each activity group contributes to the estimated *time composition* and *saddening effects*, respectively.

Table 7 shows that in both the *age*- and *fully*-*adjusted* models, the (lower) amount of time spent working contributes to a relatively higher level of experienced well-being among women compared to men, while the (higher) amount of time spent doing housework contributes to a female disadvantage in terms of experienced well-being. In addition, the lower amount of time spent in “more pleasant” activities such as leisure and self-care further contributes to the lower level of experienced well-being among women relative to men, especially when life circumstances are taken into account.

Table 8 shows the decomposition of the *saddening effect* by activity. In Panel A, we see that—when only age is controlled for—the negative *saddening effect* observed everywhere but in China is mainly driven by the fact that women have lower affective experiences during leisure or when performing housework than men. We see no consistent pattern across countries when controlling for the whole set of covariates (Panel B). Meanwhile, the positive *saddening effect* observed in China is driven by the fact that—compared to men—women have higher affective experiences during leisure and self-care activities as well as when working once individual characteristics and life circumstances are incorporated into the models. These findings suggest that specific characteristics and life circumstances of women—more than any intrinsic gender differences in activity-specific affective experiences—may be at the heart of the estimated *saddening effects*.

## Discussion

### Conclusions

Our study highlights an *age*-*adjusted* gender gap in experienced well-being in favor of men, but also shows that these gender differences weaken considerably once further individual characteristics and life circumstances are incorporated into our models. These findings suggest that at least part of the experienced well-being gap between men and women might stem from broader disadvantages of women compared to men rather than from any intrinsic “gender effect”. In particular, we find that the gender gap is largely driven by poorer average health (higher disability and self-assessed pain) and lower average economic status (permanent income quartiles) among older women when compared to older men.

We then deconstruct potential gender differences in experienced well-being into contributions of the two components of experienced well-being: time use and activity-specific net affect. Our results show that women spend more time performing housework than men, while men spend more time working and traveling. Moreover, gender differences observed for housework are generally larger than those observed for work, implying that women spend more time on work and housework combined than men. These partial associations between gender and time use are strongly statistically significant in both the *age*-*adjusted* and the *fully*-*adjusted* models. Consistent with traditional gender roles, this finding suggests that gender per se—rather than differences in individual characteristics or life circumstances between men and women—plays an important role for the large observed gender differences in time use.

Women also tend to have lower affective experiences than men across most activities when adjusting for age only. However, the inclusion of a larger set of covariates controlling for individual characteristics and life circumstances decreases this association. This attenuation in the association between gender and activity-specific net affects supports the hypothesis that other factors than gender per se are likely to be responsible for the higher activity-specific net affects of men compared to women. In particular, we find that two factors are consistently associated with net affective experiences for all activities: disability (which is negatively associated with net affect) and belonging to the highest income quartile group (which is positively associated with net affect). Our descriptive statistics show that women suffer more disability than men and belong less often to the top income quartile. These two factors thus appear to be the main drivers of the gender gap in net affective experience in favor of men.

Finally, we perform a thought experiment to disentangle the respective roles of potential *time composition* and *saddening effects* for the observed gender differences in experienced well-being. These results show that the lower experienced well-being of women compared to men of the same age is linked to their lower activity-specific net affect for all activities, and in particular for housework and leisure, irrespective of the time spent performing each activity. Perhaps somewhat surprisingly *time composition effects* contribute only marginally to the overall age-adjusted gender gap in experienced well-being, due to a compensation between the two activities considered most unpleasant, work—performed mostly by men—and housework—performed mostly by women. However, *ceteris paribus*, fully-adjusted gender differences in time use contribute to lower levels of experienced well-being of women compared to men, as the time spent in unpleasant activities by women (both work and housework) exceeds that of men with similar characteristics (in terms of disability and income in particular). Moreover, at equal levels of disability and income (among other factors), women do not appear to systematically dislike certain activities more than men (*saddening effects*). Women’s lower activity-specific net affect for all activities may thus be linked—as described above—to the conditions under which they are performed, and in particular to the higher levels of disability in women compared to men, rather than any intrinsic gender differences in net affects.

### Empirical Contributions

Our study provides new insights into gender differences in experienced well-being among older adults from different geographic and cultural settings in the developing world. Experienced well-being is an important but still relatively rarely explored dimension of emotional well-being as well as of subjective well-being more generally (National Research Council 2013). While most of the literature on subjective well-being focuses on evaluative well-being, the scarcer literature looking at emotional well-being typically considers positive and negative affective experiences separately without assessing the overall welfare implications of these different emotional experiences in terms of net affect. Moreover, given the importance of experienced well-being for the evaluation of welfare (Kahneman and Krueger 2006; Krueger and Schkade 2008), there is a surprising paucity of applied empirical work employing this measure of well-being, possibly due to the relatively low availability of DRM data, which are expensive and time-consuming to collect. In fact, to the best of our knowledge, our paper is the first study to fully explore the relationship between gender and experienced well-being in developing countries and deconstruct this relationship into its two component parts based on detailed data on both time use and activity-specific affective experiences. Due to the absence of evidence on this topic, we cannot compare our results to other studies using the same measure of subjective well-being. One notable exception is the study by Miret et al. (2012), who analyze the impact of socio-demographic characteristics on net affect using the original Day Reconstruction Method (DRM) as well as SAGE’s abbreviated version of the DRM on a sample of 1560 adults from Jodhpur (India), but without any particular focus on gender differences. These authors find that being male, living in an urban area and having a high income, are factors associated with a higher net affect, which is consistent with our own results.

We can, however, put our findings into context by comparing our results to evidence from the 2015 World Happiness Report (Fortin et al. 2015), which is one of the few studies researching the gender-specific evolution of several positive and negative emotions through the life course. Although the authors do not combine emotions into an overall net affect score, their results show generally lower levels of positive as well as higher levels of negative emotions in older women as compared to men of the same age. These data thus suggest that assessing gender differences in net affect in their context would likely yield results similar to our *age*-*adjusted* regressions, i.e. a disadvantage of older women in terms of emotional well-being. However, the 2015 World Happiness Report does not provide any analyses comparable to our *fully*-*adjusted* models, and it is thus not possible for us to assess whether the advantage for men in their assessment would disappear when individual characteristics and life circumstances are controlled for.

Our results for time use are broadly in line with the literature in the field. The evidence that women tend to perform more housework while men tend to spend more time working is remarkably similar across geographical settings and levels of wealth, and highlights the remaining importance of traditional gender roles worldwide, at least among older adults. Indeed, such gender differences in time use were found for example in Guinea by Wodon and Blackden (2006), in Ethiopia by Arbache et al. (2010), and in France, Italy, Sweden, and the USA by Anxo et al. (2011). Yet, to our knowledge, we are the first to assess the relationship between gender and activity-specific net affect, and to disentangle the respective roles of potential *time composition* and *saddening effects* for the observed gender differences in experienced well-being.

Finally, our study contributes to the methodological debate regarding the use of control variables in well-being research. On one side of the debate, Glenn (2009) claims that scholars should not control for other factors when studying the association between age and well-being. He argues that excluding control variables allows to identify the “total effects” of age on well-being, i.e., the sum of direct and any indirect effects through other variables. These total effects are, he believes, of greater importance than any potential direct effect of age holding individual characteristics and life circumstances that may change with age fixed. On the other side, some researchers argue that focusing solely on bivariate relationships is not sufficient to the understanding of the complex relationship between age and well-being, which may be mediated or confounded by other age-related differences in individual characteristics or life circumstances that may affect well-being (Blanchflower and Oswald 2008; Blanchflower and Oswald 2009). In this context, we perform both *age*-*adjusted* comparisons of subjective well-being between men and women as well as *fully*-*adjusted* regressions of subjective well-being on gender that also account for gender differences in health status, socio-demographic characteristics and community participation in the same dataset. Our *age*-*adjusted* models, on the one hand, can provide evidence regarding potential advantages or disadvantages of women in terms of their experienced well-being compared to their male counterparts. These analyses are especially important for a descriptive assessment of overall gender inequalities in experienced well-being, as well as for the targeting of potential policies and interventions aimed at mitigating them. The *fully*-*adjusted* analyses, on the other hand, account for potential gender differences in other individual characteristics and life circumstances. These may at least in part mediate the *age*-*adjusted* relationship between gender and experienced well-being and thus isolate the partial association of gender with experienced well-being *ceteris paribus*. In addition, these analyses highlight potential policy levers related to gender differences in individual characteristics and life circumstances that may be helpful in alleviating gender differences in experienced well-being. Our analyses confirm our working hypothesis that the results obtained through the two approaches provide different but equally important views on the association between gender and experienced well-being and should therefore be seen as complementary.

### Practical Implications

We are facing a situation without precedent: by 2050, it is estimated that there will be more than twice as many persons over the age of 65 than under the age of 5 (United Nations 2019a). This rapid demographic transition is raising important issues not just in industrialized countries but worldwide, as an increasingly large proportion of the older population is living in low- and middle-income countries. In order to meet the post-2015 sustainable development agenda goal of ensuring healthy lives and promoting well-being for everyone at all ages (United Nations 2019b) as well as to enable healthy aging for everybody (World Health Organization 2015), social and health systems worldwide must find effective ways to respond to the needs of older adults. In the near future, increasing the healthspan (i.e., the time that an individual is able to live in good health) as well as quality of life of older adults, and not merely preventing deaths, will be a key objective of health and social interventions. The scarcity of knowledge regarding the drivers of older persons’ experienced well-being—especially in low- and middle-income countries—must therefore urgently be addressed in order to construct effective responses to global population aging using evidence-based policies. Due to women’s higher longevity, a majority of older adults is female, especially at very advanced ages. Moreover, while women generally constitute a vulnerable group, they may be especially at risk in older age. For example, older women tend to suffer from more chronic health conditions than men of the same age, be poorer, and have lower access to health care services (World Health Organization 2015). However, compared to men, women may benefit from stronger family support from adult children, and may suffer in lower numbers from the negative impacts of role disruption at retirement (Knodel and Ofstedal 2003). It is thus crucial to understand whether and how these objective circumstances translate into subjective well-being differences in old age in order to design policies to address them.

Our paper yields information that is potentially useful to policy makers. First, we document that the gender gap in emotional well-being is pervasive. In particular, we show that much of the gender gap in experienced well-being which disadvantages women relative to men can be linked to gender differences in activity-specific affective experiences. This finding suggests that just moving toward a more equitable time repartition within households may not be sufficient to close the existing gender gaps in experienced well-being. Moreover, we show that gender differences in individual characteristics and life circumstances, notably disability and income, are key factors underlying the experienced well-being gap in favor of men. These findings suggest that policies, such as female-targeted campaigns for the early prevention of disability, and increasing entitlement programs for older women, may prove useful in improving women’s experienced well-being at older ages. In addition, the empowerment of older women can be encouraged by life-long interventions, such as the promotion of equitable workforce participation, the implementation of compulsory social contributions, and the distribution of non-contributory social pensions at all ages. Finally, health promotion and disease prevention interventions targeted not only towards older populations but also towards younger individuals have the potential of keeping older adults in good health for much longer in the future. These policies should complement more general efforts to improve well-being in older age by improving health and social support systems as well as addressing the social determinants of health.

### Limitations and Future Research Directions

To the best of our knowledge, we are the first to assess and deconstruct gender differences in experienced well-being using DRM data from a large-scale multi-country survey effort in the developing world. Performing our analyses on harmonized data from different countries allows us to document robust associations across different cultural contexts and geographic regions. In addition, our study shows the added value of using DRM-based data to explore the respective roles of time use and activity-specific affective experiences for explaining gender differences in experienced well-being. Nevertheless, our estimated partial associations cannot be interpreted as causal due to potential issues of confounding, reverse causation and selection into activities. Estimating average activity-specific net affects only using data from individuals who actually perform these activities may be particularly problematic in this regard. More research is thus needed to address these limitations, in order to allow inference of a causal relationship, perhaps in the context of a structural model for time use. Finally, while our study focuses exclusively on older adults, it would be worthwhile to evaluate how the relationship between gender and experienced well-being evolves over the life course. In spite of these limitations, our study makes a valuable contribution by documenting and deconstructing gender differences in experienced well-being among older adults from different developing country setting and highlighting key individual characteristics and life circumstances beyond gender itself that may help explain gender differences in experienced well-being.

## Appendix 1

See Table 9.

**Table 9:** Partial association of gender with experienced well-being *U* for individuals aged 50+

	Ghana	India	China	South Africa	Russia
Female	**− **0.0536	**− **0.0822	0.0070	**− **0.0328	**− **0.1063
Age 60–69	0.1798***	0.1257***	0.0880***	0.3629***	**− **0.0140
Age 70–79	0.2251***	0.2445***	0.2131***	0.2616**	0.1667
Age 80+	0.2645***	0.1411	0.3380***	0.4952***	0.2362*
Rural	**− **0.0705	**− **0.0510	**− **0.2173***	**− **0.059	0.0662
Married	**− **0.0055	**− **0.0789	0.0490	0.1766**	**− **0.1148
Number of adults in the household	**− **0.0387***	0.0084	**− **0.0327***	**− **0.0884**	0.0468
Number of children in the household	**− **0.0008	0.0086	0.0039	0.0422	**− **0.0284
Enough money to meet one’s needs	**− **0.1744***	0.0565	0.2385***	**− **0.0666	0.1979**
Education (nb of years)	0.0023	0.0047	0.0026	0.0072	**− **0.0085
Working	0.0904***	**− **0.0928***	**− **0.0212***	**− **0.0446***	**− **0.2615***
Q2: permanent income	0.0766	0.0697	0.0870**	0.0607	0.2823***
Q3: permanent income	0.2054***	0.0321	0.2259***	0.1911*	0.2160**
Q4: permanent income	0.2491***	0.2144***	0.2459***	0.1593	0.3887***
Victim of a violent crime (last 12 m)	0.0413	**− **0.0735	0.0473	0.0733	**− **0.0123
Community involvement	0.0830***	**− **0.0473**	0.0381***	**− **0.0006	**− **0.0933**
Trust	0.0070	0.0046	0.0076	**− **0.0391	**− **0.0408
Safety	0.0432*	0.0948***	0.1327***	**− **0.0155	0.1092***
WHO disability score	**− **0.1038***	**− **0.2578***	**− **0.0523***	**− **0.1722***	**− **0.2169***
Self-assessed pain	0.0253	**− **0.0466*	**− **0.0582***	**− **0.1145**	**− **0.0652
Constant	**− **0.3721**	0.4718**	**− **0.9978***	0.5838**	0.5656
Number of observations	3031	4833	9106	2005	2513

Standard errors are computed using 250 bootstrap replications. Sample weights are applied. The entries in each column are country-specific average partial effects of gender on experienced well-being. Average partial effects are based on a linear regression (Eq. (7))

**p* < 0.10, ***p* < 0.05, ****p* < 0.01

## Appendix 2

See Table 10.

**Table 10:** Partial association of gender with time shares τ_a_ for individuals aged 50+

	Ghana	India	China	South Africa	Russia
*Work*					
Female	**− **0.0321**	**− **0.0553***	**− **0.0505***	**− **0.0560***	**− **0.0431
Age 60–69	**− **0.0352**	**− **0.0132	**− **0.0199**	**− **0.0323*	0.0168
Age 70–79	**− **0.0428***	**− **0.0650***	**− **0.0686***	**− **0.0556**	**− **0.0082
Age 80+	**− **0.0910***	**− **0.0519**	**− **0.1212***	**− **0.0126	**− **0.0931
Rural	**− **0.0057	**− **0.0067	0.0507***	0.0044	0.0252
Married	**− **0.0217	**− **0.0087	**− **0.0061	**− **0.0141	0.0768**
Number of adults in the household	**− **0.0001	0.0033	0.0202***	0.0057	**− **0.0291***
Number of children in the household	**− **0.0009	**− **0.0019	**− **0.0128*	0.0045	0.0329**
Enough money to meet one’s needs	0.0320**	**− **0.0059	0.0117	**− **0.0051	0.0021
Education (years)	**− **0.0007	**− **0.0008	**− **0.0030**	**− **0.0045**	**− **0.0005
Working	0.2929***	0.1567***	0.1722***	0.1493***	0.2389***
Q2: permanent income	**− **0.0041	0.0143	**− **0.0003	0.0201	0.0329
Q3: permanent income	**− **0.0284*	0.0028	**− **0.0209*	0.0284	0.0070
Q4: permanent income	**− **0.0066	**− **0.0343**	0.0044	0.0503**	**− **0.0043
Victim of a violent crime (last 12 m)	**− **0.0262	0.0250	**− **0.0150	**− **0.0430	0.0221
Community involvement	0.0153**	0.0089*	**− **0.0033	0.0145*	0.0421***
Trust	**− **0.0064	0.0006	**− **0.0103***	**− **0.0016	**− **0.0188*
Safety	0.0012	**− **0.0092**	0.0001	**− **0.0130*	**− **0.0200*
WHO disability score	0.0214***	**− **0.0194***	**− **0.0210***	**− **0.0096	**− **0.0236
Self-assessed pain	**− **0.0126*	**− **0.0052	**− **0.0001	**− **0.0101	**− **0.0265*
*Housework*					
Female	0.1162***	0.2194***	0.1590***	0.1204***	0.2168***
Age 60–69	0.0098	**− **0.0139***	**− **0.0020	0.0173**	0.0267
Age 70–79	0.0019	**− **0.0432***	**− **0.0245***	0.0036	0.0068
Age 80+	**− **0.0117	**− **0.0792***	**− **0.0352***	**− **0.0033	0.0959
Rural	**− **0.0001	0.0311***	0.0255***	0.0164**	**− **0.0023
Married	0.0147**	0.0394***	0.0141***	**− **0.0134*	0.0299
Number of adults in the household	**− **0.0005	**− **0.0037***	**− **0.0110***	0.0000	0.0039
Number of children in the household	**− **0.0006	0.0003	0.0500***	0.0059**	**− **0.0117
Enough money to meet one’s needs	**− **0.0135**	0.0099*	**− **0.0135***	**− **0.031***	0.0071
Education (years)	**− **0.0030***	0.0013*	**− **0.0007**	0.0045***	**− **0.0042**
Working	**− **0.0146*	**− **0.0071	**− **0.0321***	**− **0.0869***	**− **0.1096***
Q2: permanent income	0.0164**	**− **0.0247***	**− **0.0171***	**− **0.0338***	0.0107
Q3: permanent income	0.0155**	**− **0.0052	**− **0.0306***	**− **0.0619***	0.0308
Q4: permanent income	0.0038	**− **0.0246***	**− **0.0373***	**− **0.0177*	0.0000
Victim of a violent crime (last 12 m)	0.0188	0.0061	0.0118	**− **0.0092	**− **0.0848
Community involvement	**− **0.0081**	0.0127***	0.0032**	**− **0.0018	**− **0.0029
Trust	0.0016	**− **0.0009	**− **0.0040***	**− **0.0114***	**− **0.0078
Safety	**− **0.0078***	**− **0.0100***	**− **0.0146***	**− **0.0017	0.0070
WHO disability score	**− **0.0415***	**− **0.0083**	**− **0.0219***	**− **0.0241***	**− **0.0310*
Self-assessed pain	0.0103***	**− **0.0007	0.0033*	0.0009	0.0162*
*Travel*					
Female	**− **0.0200***	**− **0.0269***	**− **0.0093***	**− **0.0143**	**− **0.0385*
Age 60–69	**− **0.0126**	0.0005	**− **0.0047	**− **0.0151*	**− **0.0331
Age 70–79	**− **0.0063	0.0031	**− **0.0019	0.0032	**− **0.0314
Age 80+	0.0020	0.0014	**− **0.0108	**− **0.0006	**− **0.1399
Rural	**− **0.0010	0.0146*	**− **0.0154***	**− **0.0038	0.0477**
Married	**− **0.0056	**− **0.0041	**− **0.0091**	**− **0.0016	**− **0.0405
Number of adults in the household	**− **0.0025*	**− **0.0020*	0.0007	0.0033*	0.0086
Number of children in the household	0.0008	0.0027**	0.0032	**− **0.0122***	**− **0.0149
Enough money to meet one’s needs	0.0009	0.0113**	**− **0.0019	**− **0.0089	**− **0.0009
Education (years)	0.0011*	0.0017**	0.0002	**− **0.0017**	**− **0.0010
Working	0.0191**	**− **0.0056	**− **0.0031	**− **0.0023	**− **0.0173
Q2: permanent income	**− **0.0065	0.0015	**− **0.0022	0.0118	**− **0.0377*
Q3: permanent income	0.0011	**− **0.0053	**− **0.0066*	0.0079	**− **0.0446**
Q4: permanent income	**− **0.0023	**− **0.0100	**− **0.0089**	0.0188*	0.0068
Victim of a violent crime (last 12 m)	0.0105	0.0093	0.0108	**− **0.0093	0.0947
Community involvement	0.0080**	0.0043*	0.0028**	0.0111***	**− **0.0140
Trust	**− **0.0018	0.0024	**− **0.0041***	0.0039	**− **0.0048
Safety	0.0000	0.0081***	0.0021	**− **0.0028	0.0070
WHO disability score	**− **0.0104***	**− **0.0048	0.0006	**− **0.0189***	**− **0.0446***
Self-assessed pain	0.0033	**− **0.0011	**− **0.0019	0.0082	0.0150*
*Leisure*					
Female	**− **0.0703***	**− **0.1129***	**− **0.0891***	**− **0.0617***	**− **0.1074***
Age 60–69	0.0384**	0.0150	0.0236**	0.0322	0.0073
Age 70–79	0.0460**	0.0807***	0.0857***	0.0699**	0.0354
Age 80+	0.0911***	0.0982***	0.1451***	0.0161	0.1464**
Rural	0.0155	**− **0.0214	**− **0.0402***	**− **0.0091	**− **0.0674**
Married	**− **0.0102	**− **0.0265*	**− **0.0026	0.0403*	**− **0.0352
Number of adults in the household	0.0016	0.0018	**− **0.0079*	**− **0.0084	0.0095
Number of children in the household	**− **0.0011	**− **0.0042	**− **0.0411***	0.0029	0.0009
Enough money to meet one’s needs	**− **0.0314**	**− **0.0289**	0.0055	0.0342	**− **0.0144
Education (years)	0.0043***	**− **0.0019	0.0035***	0.0029	0.0058*
Working	**− **0.2406***	**− **0.1328***	**− **0.1380***	**− **0.0686***	**− **0.1479***
Q2: permanent income	**− **0.0007	0.0180	0.0231*	0.0077	0.0151
Q3: permanent income	**− **0.0088	0.0036	0.0513***	0.0493	**− **0.0036
Q4: permanent income	0.0123	0.0730***	0.0375***	**− **0.0425	**− **0.0237
Victim of a violent crime (last 12 m)	0.0221	**− **0.0555*	0.0167	0.0303	**− **0.0173
Community involvement	**− **0.0188**	**− **0.0205***	**− **0.0034	**− **0.0285***	**− **0.0157
Trust	**− **0.0137*	**− **0.0070	0.0189***	0.0137	0.0338***
Safety	0.0184***	0.0071	0.0112***	0.0220*	0.0082
WHO disability score	0.0355***	0.0101	0.0346***	0.0346***	0.0595***
Self-assessed pain	0.0007	0.0105	0.0003	0.0081	**− **0.0005
*Selfcare*					
Female	0.0062	**− **0.0244***	**− **0.0101***	0.0116	**− **0.0277
Age 60–69	**− **0.0004	0.0116	0.0031	**− **0.0021	**− **0.0177
Age 70–79	0.0013	0.0243**	0.0093*	**− **0.0211	**− **0.0026
Age 80+	0.0097	0.0314*	0.0222**	0.0004	**− **0.0093
Rural	**− **0.0088	**− **0.0175	**− **0.0206***	**− **0.0078	**− **0.0031
Married	0.0228*	**− **0.0002	0.0037	**− **0.0111	**− **0.0310
Number of adults in the household	0.0015	0.0005	**− **0.0021	**− **0.0006	0.0070*
Number of children in the household	0.0018	0.0032*	0.0007	**− **0.0011	**− **0.0073
Enough money to meet one’s needs	0.0119	0.0136*	− 0.0018	0.0107	0.0060
Education (years)	− 0.0017**	− 0.0003	0.0000	− 0.0012	− 0.0002
Working	− 0.0569***	− 0.0113	0.0009	0.0085	0.0358**
Q2: permanent income	− 0.0051	− 0.0090	− 0.0036	− 0.0058	− 0.0210
Q3: permanent income	0.0206	0.0041	0.0068	− 0.0237	0.0104
Q4: permanent income	− 0.0072	− 0.0041	0.0044	− 0.0089	0.0212
Victim of a violent crime (last 12 m)	− 0.0251	0.0151	− 0.0244**	0.0311	− 0.0147
Community involvement	0.0036	− 0.0054	0.0007	0.0046	− 0.0095
Trust	0.0204***	0.0048	− 0.0005	− 0.0046	− 0.0024
Safety	− 0.0119***	0.0040	0.0012	− 0.0044	− 0.0022
WHO disability score	− 0.0049	0.0224***	0.0077***	0.0181**	0.0397***
Self-assessed pain	− 0.0017	− 0.0035	− 0.0016	− 0.0071	− 0.0042

Standard errors are computed using 250 bootstrap replications. Sample weights are applied. The entries in each column are country-specific average partial effects of gender on experienced well-being. Average partial effects are based on a multivariate fractional logit model (Eq. (10) and (11))

**p* < 0.10, ***p* < 0.05), ****p* < 0.01

## Appendix 3

See Table 11.

**Table 11:** Partial association of gender with activity-specific net affects ua for individuals aged 50+

	Ghana	India	China	South Africa	Russia
*Work*					
Female	− 0.0763	0.0992	0.1088**	0.1865	0.0187
Age 60–69	0.1248	0.2284**	0.1265**	0.7241***	− 0.0061
Age 70–79	0.2193**	− 0.0573	0.4486***	0.4403	− 0.0475
Age 80+	− 0.1200	0.1372	0.5598***	1.0891***	0.2811
Rural	− 0.0273	− 0.0349	− 0.6747***	0.1112	0.3087**
Married	0.0155	− 0.3465***	0.0095	0.3492**	− 0.1487
Number of adults in the household	− 0.0660***	0.0532**	− 0.0499	− 0.1741**	0.0089
Number of children in the household	− 0.0019	− 0.0426	0.0920*	0.2322***	− 0.0277
Enough money to meet one’s needs	− 0.1142	0.1984**	0.1916***	− 0.1350	0.0000
Education (years)	− 0.0046	0.0198*	− 0.0049	− 0.0148	− 0.0121
Working	0.5211**	− 0.0710	0.1476**	0.2891	− 0.2929***
Q2: permanent income	0.0898	0.0063	0.3135***	0.0924	0.4374**
Q3: permanent income	0.1756	0.0068	0.4739***	0.2120	0.4521***
Q4: permanent income	0.3426**	0.0339	0.4059***	0.5226**	0.5586***
Victim of a violent crime (last 12 m)	− 0.0515	0.0131	0.1251	− 0.4420	− 0.8119*
Community involvement	0.1250***	− 0.0947*	0.0229	0.0951	− 0.0328
Trust	0.0122	0.0514	0.0937***	− 0.0886	− 0.1522**
Safety	− 0.0114	0.1192**	0.1761***	− 0.0994	0.0422
WHO disability score	− 0.1324**	− 0.2942***	− 0.0069	− 0.5538***	− 0.4574***
Self-assessed pain	0.0350	− 0.0822	− 0.0776***	− 0.1691	0.0973
*Housework*					
Female	0.0457	− 0.1128	0.0113	− 0.0703	− 0.1521
Age 60–69	0.2003***	0.1662**	0.0801**	0.3028***	− 0.2722*
Age 70–79	0.1382	0.2549**	0.2922***	0.4140***	0.0435
Age 80+	− 0.0083	− 0.3714	0.3009***	0.4865**	0.0792
Rural	− 0.0100	− 0.0562	− 0.2642***	− 0.0989	0.0181
Married	0.0095	− 0.0860	0.0899**	− 0.0002	− 0.1215
Number of adults in the household	0.0124	0.0153	− 0.0544***	− 0.0527	0.0423
Number of children in the household	− 0.0196	0.0194	0.0021	− 0.0080	− 0.0648
Enough money to meet one’s needs	− 0.1539**	0.1263*	0.2286***	0.0268	0.2782**
Education (years)	− 0.0038	0.0027	0.0030	− 0.0002	− 0.0046
Working	− 0.0417	− 0.0487	0.1269***	− 0.0752	− 0.2243*
Q2: permanent income	0.1084	0.0993	0.0486	0.0017	0.3702**
Q3: permanent income	0.0451	0.0537	0.1936***	0.1372	0.3077*
Q4: permanent income	0.1818*	0.3003***	0.2176***	0.2107	0.3825**
Victim of a violent crime (last 12 m)	− 0.1974	− 0.3811*	0.0572	0.0381	− 0.0484
Community involvement	0.0291	− 0.0197	0.0586***	0.0572	0.0091
Trust	− 0.0123	0.0354	0.0052	− 0.0709*	− 0.0497
Safety	0.1239***	0.0982***	0.1425***	− 0.0307	0.0830
WHO disability score	− 0.1271**	− 0.3438***	− 0.0721***	− 0.2435***	− 0.3618***
Self-assessed pain	− 0.0213	− 0.0704*	− 0.0636***	0.0013	− 0.0801
*Travel*					
Female	− 0.0288	− 0.2401**	0.0038	0.0305	− 0.2403
Age 60–69	0.0369	0.1425	0.1043	0.3550***	0.4752**
Age 70–79	0.1075	0.2042	0.2782***	0.3753**	0.0113
Age 80+	0.1839	− 0.0283	0.3811**	0.7522***	− 0.5925
Rural	− 0.1616**	0.1512	− 0.3987***	− 0.3342**	0.5754***
Married	− 0.0182	− 0.0604	− 0.0151	0.3378***	− 0.4792**
Number of adults in the household	− 0.0288*	0.0073	− 0.0455	0.0022	0.0716
Number of children in the household	0.0153	0.0092	0.0569	− 0.1037	− 0.1573
Enough money to meet one’s needs	0.0506	0.1955*	0.0511	0.1620	− 0.0587
Education (years)	− 0.0036	0.0026	− 0.0050	− 0.0210*	− 0.0323
Working	− 0.0295	− 0.2241**	0.0459	− 0.0944	0.1305
Q2: permanent income	0.0748	0.1172	0.0065	− 0.3132**	0.2347
Q3: permanent income	0.0733	0.1843	0.0677	− 0.3442**	− 0.0297
Q4: permanent income	0.3007***	0.4160***	− 0.0189	− 0.3107	0.2487
Victim of a violent crime (last 12 m)	0.0341	0.0293	− 0.0946	0.1019	0.9509
Community involvement	0.1326***	0.0150	0.0106	0.1400*	− 0.1392
Trust	0.0365	− 0.0407	− 0.0040	0.0174	0.0277
Safety	0.0692**	0.1322***	0.2005***	0.0667	0.1229
WHO disability score	− 0.1224**	− 0.2163**	− 0.0280	− 0.1153	− 0.5335***
Self-assessed pain	0.0204	− 0.1488***	− 0.1388***	− 0.1900**	− 0.0845
*Leisure*					
Female	− 0.0777	− 0.0618	0.0477**	− 0.1283**	0.0429
Age 60–69	0.1378**	0.0619	0.0822***	0.3221***	0.0754
Age 70–79	0.1883**	0.1716***	0.1419***	0.2765**	0.3112***
Age 80+	0.3221***	0.1012	0.2685***	0.3948***	0.4449***
Rural	− 0.0933**	0.0189	− 0.1234***	− 0.0561	− 0.0527
Married	− 0.0185	− 0.0571	0.0564	0.1642**	0.1708**
Number of adults in the household	− 0.0406**	0.0017	− 0.0135	− 0.0781	0.0069
Number of children in the household	0.0104	0.0168	0.0108	0.0273	0.0933*
Enough money to meet one’s needs	− 0.1235**	− 0.0155	0.0896**	− 0.0301	0.2165***
Education (years)	− 0.0036	0.0050	0.0013	0.0073	− 0.0094
Working	0.2312***	0.0065	0.0378	0.0421	− 0.0006
Q2: permanent income	0.0836	0.0883	0.0702**	0.0761	0.2031**
Q3: permanent income	0.2803***	0.0348	0.1696***	0.1842	0.0978
Q4: permanent income	0.2168***	0.1819***	0.1888***	0.1122	0.2511**
Victim of a violent crime (last 12 m)	0.1182	0.0402	0.0187	0.0647	0.1389
Community involvement	0.0795***	− 0.0422*	0.0387***	− 0.0008	0.0041
Trust	− 0.0145	− 0.0093	− 0.0128	− 0.0225	− 0.0701**
Safety	0.0361	0.0742***	0.1104***	− 0.0522	0.0595
WHO disability score	− 0.1173***	− 0.2386***	− 0.0910***	− 0.0978**	− 0.1559***
Self-assessed pain	0.0500*	− 0.0396	− 0.0339**	− 0.1121***	− 0.0722
*Selfcare*					
Female	0.0343	− 0.0193	0.0472**	0.0528	− 0.1246
Age 60–69	0.0651	0.0761*	0.0729**	0.2424***	− 0.0902
Age 70–79	0.0344	0.1749***	0.2380***	0.1107	0.1164
Age 80+	0.0962	0.1656	0.3366***	0.2683	0.1382
Rural	− 0.0514	− 0.0856*	− 0.1685***	− 0.0588	− 0.1389
Married	− 0.0184	− 0.0243	0.1689***	0.1265*	− 0.1013
Number of adults in the household	− 0.0296**	0.0006	− 0.0427***	− 0.0702***	0.0659**
Number of children in the household	0.0068	0.0129	0.0657**	0.0336	0.0743
Enough money to meet one’s needs	− 0.1843***	− 0.0126	0.0930**	− 0.0575	0.2023**
Education (years)	− 0.0019	0.0095	0.0017	0.0144**	− 0.0053
Working	0.1689***	− 0.0446	0.0297	− 0.0800	− 0.1164
Q2: permanent income	0.1021*	0.0121	0.0208	0.0157	0.2056*
Q3: permanent income	0.2301***	− 0.0257	0.1232***	0.1546*	0.2641**
Q4: permanent income	0.2536***	0.1385*	0.1800***	0.2298**	0.3831***
Victim of a violent crime (last 12 m)	0.0497	0.1649	0.1886**	− 0.0554	0.3403***
Community involvement	0.0548**	0.0000	0.0197	− 0.0024	− 0.0420
Trust	− 0.0367*	− 0.0145	0.0062	0.0060	− 0.0691*
Safety	0.0278	0.0812***	0.1258***	0.0162	0.0041
WHO disability score	− 0.0718***	− 0.2435***	− 0.0964***	− 0.1600***	− 0.2556***
Self-assessed pain	0.0463*	0.0172	− 0.0290*	− 0.0327	− 0.0776

Standard errors are computed using 250 bootstrap replications. Sample weights are applied. The entries in each column are country-specific average partial effects of gender on Activity-Specific Net Affects. Average partial effects are based on a linear regression (Eq. (13))

**p* < 0.10, ***p* < 0.05, ****p* < 0.01

## Footnotes

1 Women represented 86 percent of individuals out of the labor force and 60 percent of unpaid workers in 2018 (World Bank Group 2018).

2 Occasionally, researchers further explicitly distinguish a third dimension of subjective well-being, eudaimonic well-being, which, however, shares many characteristics with evaluative well-being and is, therefore, also often subsumed in the broader category of evaluative well-being (OECD 2013).

3 Miret et al. (2012) show that the data obtained using this tool are largely comparable to those obtained using a full DRM instrument.

4 **Work:** working, subsistence farming. **Housework:** preparing food, doing housework, watching children, shopping, providing care to someone. **Travel**: walking somewhere, traveling by bicycle, traveling by car/bus/train. **Leisure**: rest (including tea/coffee break), chatting with someone, playing (including cards/games), reading, listening to radio, watching TV, exercising or leisure walk, other leisurely activity. **Self-care**: grooming or bathing (self), eating, religious activity, intimate relations/sex.
